# Roles of Pho regulon in bacterial pathogenicity

**DOI:** 10.1080/21505594.2025.2545559

**Published:** 2025-08-13

**Authors:** Jing Yang, Min Wang, Yi Wang, Rui Qiang, Qiyuan Jin, Chenhao Zhao, Qi Chen, Mingxiao Han, Xin Ma, Haifang Zhang

**Affiliations:** a Department of Clinical Laboratory, The Second Affiliated Hospital of Soochow University, Suzhou, China; b MOE Key Laboratory of Geriatric Diseases and Immunology, Soochow University, Suzhou, China

**Keywords:** Pho regulon, phosphate homeostasis, bacterial pathogenicity

## Abstract

Bacterial infections are a leading cause of global health loss. The prerequisites for bacteria to colonize and establish systemic infection in the host are environmental adaptability and the expression of virulence factors. Phosphorus constitutes the fifth most important element in terms of its cellular content, which is pivotal for DNA replication, metabolism, signal transmission, and microbial cell composition. The phosphate (Pho) regulon is a well-established unique mechanism in response to inorganic phosphate (Pi) starvation. The Pho regulon is strongly related to bacterial pathogenicity, except for the simple regulatory system for Pho balance. The PhoBR two-component regulatory system of the Pho regulon has documented the effects of affecting virulence in microbes. This review emphasizes the impact of the absence of PhoB and virulence-related gene regulation by PhoB on pathogenicity in common pathogens of *Escherichia coli*, *Pseudomonas aeruginosa*, *Salmonella* enterica serovar *Typhimurium*, *Vibrio cholerae*, etc. Collectively, Pho regulon is a regulatory network connecting Pho homeostasis with bacterial virulence. This study may offer valuable information for understanding the regulation of bacterial virulence by the Pho regulon, providing novel insights for the development of antimicrobial strategies.

## Introduction

Bacteria must adapt to fluctuations in microenvironmental conditions such as nutrients, acidity, and temperature for survival and infection. The carbon: nitrogen: phosphorus ratio is a crucial metabolic pathway that supplies nutrients for all living organisms to grow and proliferate [1]. Acting as an essential component for cellular life, phosphorus, present from different origins (inorganic, bioorganic), has crucial roles in bacterial heredity, energy metabolism, and intracellular signaling, which is also involved in forming nucleic acids, ATP, and phospholipids. Phosphorus acquired in the form of inorganic phosphate (Pi) is one of the main sources of bacterial phosphorus. The phosphate (Pho) regulon, involving a large number of coregulated genes whose expression is controlled by the environmental Pi level, is a complex and accurate signal transduction system to deal with the shortage of phosphorus nutrients. Pho regulon, identified and studied for the first time in *Escherichia coli* (*E. coli*) [2], consists of *phoBR*, *pstSCAB* and *phoU*, which encode a two-component regulatory system (TCRS) PhoBR, Pho-specific transporter PstSCAB, and auxiliary protein PhoU [3]. *E. coli* contains two major Pi transport systems, the low-affinity Pi transporter (Pit) and the high-affinity Pho-specific transporter system (PstSCAB) [4]. In the context of sufficient Pi, bacteria rely on the low-affinity Pit to maintain cytoplasmic Pi [4–6]. However, that transporter can no longer meet cellular demand under Pi-limited condition (Pi concentration falls below 4 μM), necessitating the PstSCAB that is dependent on the TCRS PhoBR, a key signaling pathway adapting to Pi scarcity in *E. coli* [7,8]. PhoBR was reported to respond to intracellular Pi signal according to recent studies of *Salmonella enterica serovar Typhimurium* (*S. typhimurium*) [9–11].

Serving as a TCRS, PhoBR is a typical signal-sensing response system in bacteria that constitutes a dominant form of genes control in response to changes [12]. It is composed of a response regulator (RR) that activates or represses the transcription of specific genes and an inner membrane histidine kinase (HK) sensor protein [13]. As a RR in PhoBR TCRS, PhoB can bind to a pho box, a 22-base pair sequence with two 11-base pair direct repeat units, in the promoter region to regulate the expression of the Pho regulon genes [14]. The pho box was incidentally identified in the promoter region of some virulence genes in *phoB*-mutant strain and revealed that PhoB could regulate their expression, highlighting the additional roles of the PhoBR TCRS in bacterial pathogenicity and host-pathogen interactions [3,8,15–17].

There have been extensive studies regarding the role of Pho regulon in pathogenic bacteria such as *E. coli*, *Pseudomonas aeruginosa* (*P. aeruginosa*), *S. typhimurium, Vibrio cholerae (V. cholerae)*, *Klebsiella pneumoniae* (*K. pneumoniae*), and *Edwardsiella tarda* (*E. tarda*) [8,18–20]. Corresponding findings underscore the predominant impact of the Pho regulon on bacterial virulence factors, requiring further in-depth understanding to establish more specific relationship between Pho regulation and bacterial pathogenicity. Accordingly, the present review was designed to explore the relationship between the Pho regulon and bacterial pathogenicity, with a focus on the regulation of virulence by PhoB.

## PstSCAB-PhoU-PhoBR signaling system

### The PhoBR TCRS

**Figure 1.**
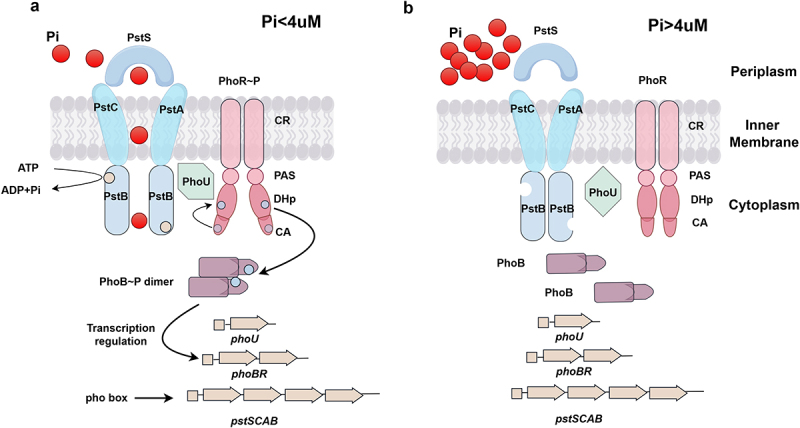
Induction of PstSCAB-PhoU-PhoBR signaling system for Pho regulon gene transcription and Pi acquisition under Pi starvation.

The TCRS is a widely distributed signal sensing and stress response system in bacteria. PhoBR TCRS can regulate the expression of Pho regulon genes to adapt to Pi- limited environments [7,17,21]. This TCRS is composed of the inner membrane sensor histidine kinase PhoR and response regulator PhoB [22]. PhoB contains an N-terminal receiver domain and a C-terminal DNA-binding domain (DBD) to participate in signal transformation and gene transcription (Figure 1). PhoR features a cytoplasmic charged region, Per-Arnt-Sim (PAS) domain, dimerization and histidine phosphotylation (DHp) domain, and catalytic active/ATP-binding (CA) domain (Figure 1). Among these, the CA domain contains a conserved ATP-binding pocket that confers PhoR with autokinase, phosphotransferase, and phosphatase activity [21]. In Pi-limited environments, PhoR can autophosphorylate the histidine residue, leading to subsequent transfer of the phosphoryl group to the aspartic acid residue of PhoB [22,23]. As a result, the phosphorylated PhoB dimerize and bind to the pho box, leading to the activation of gene transcription by recruiting RNA polymerase [3,24].

(a) When environmental Pi levels fall below 4 μM, PhoR autophosphorylates. Then the Pho group is transferred to the aspartic acid residue on PhoB. The phosphorylated PhoB dimerizes, binds to pho box and activates the transcription of Pho regulon genes. During the transport of Pi from the periplasm to cytoplasm, PstS attaches Pi and presents it to PstC and PstA, then the outward-facing conformation is converted into the inward-facing conformation through the cooperation of PstC, PstA, and PstB. PhoU serves as a bridging protein between PhoR and PstSCAB. (b) When the Pi levels exceed 4 μM, the PstSCAB-PhoU-PhoBR signaling system will not be activated.

### The transporter PstSCAB

The Pho-specific transporter PstSCAB facilitates the acquisition of Pi by functioning as an ATP-binding cassette (ABC) transporter. PstS is a periplasmic protein that binds Pi with high affinity. PstC and PstA are inner-membrane channel proteins to benefit the entering of Pi into the cytoplasm [25,26]. PstB is the dimeric nucleotide-binding domain (NBD) where ATP is bound and hydrolyzed to supply the energy needed for Pi transport from the periplasm to the cytoplasm [27]. Previously, PstSCAB transporter was reported to operate through a mechanism analogous to the maltose ABC transporter (MalFGK), a model to study the mechanism of ABC transporters in *E. coli*, the substrate transport was achieved by binding and hydrolyzing the conformational changes initiated by ATP in the NBD domain [28]. Q160 and E179 of PstB were found to correspond to Q140 and E159 of MalK, and that MalK Q140 K mutant and MalK E159 Q mutant were considered to maintain open and closed conformation [28,29]. Moreover, with the construction of *pstB*Q160K and *pstB*E179Q mutant, the research predicted that *pstB*Q160K stabilizing the inward-facing conformation, and the *pstB*E179Q mutation stabilizing the outward-facing conformation [28], the transition between these two conformations may represent the mechanism by which Pi is transported (Figure 1).

### The auxiliary protein PhoU

PhoU connects the PhoR and PstSCAB transporter, which has been discovered through the bacterial two-hybrid analysis, to enable the interaction with the PAS domain of PhoR and PstB, with stronger interaction of PhoU-PhoR than PhoU-PstB [30]. However, so far, there is a poor understanding of the impact of PhoU-PstB interaction. A prior study from *S. typhimurium* revealed that the interaction of PhoU-PhoR PAS domains participated in suppressing Pho regulon gene expression in high-Pi environments, and the interaction of the PhoU Arg184 residue-PhoR HK domain involved the activation of PhoR HK autophosphorylation to promote the activation of PhoBR under low-Mg^2+^ condition [31]. Related research showed that PhoBR may response to the low cytoplasmic Pi in low extracytoplasmic Mg^2+^ condition in *S. typhimurium* [10,11,32]. Cellular ATP is essential for ribosome synthesis, and Mg^2+^ is required for ribosomes stabilization [33]. The PhoPQ was activated for MgtA, MgtB and MgtC (Mg^2 +^ transporters) transcription in low extracytoplasmic Mg^2+^ condition in *S. typhimurium*, which led to decreased cytoplasmic Mg^2+^ for ribosomes stabilization in protein synthesis process [10]. As the decrease of free cytoplasmic Mg^2+^, ribosome subunits failed to associate efficiently, coupled with retarded translation and reduced ATP consumption, which prevented the Pi liberation from ATP into the cytosol, leading to a reduction in free cytoplasmic Pi that is proposed to activate the PhoBR [10]. Furthermore, in their study, following 5 h of growth in low Mg^2+^ medium, cytoplasmic Pi levels were confirmed to be lower in wild type *S. typhimurium* than the *mgtC* mutant, and *mgtC* expression from a heterologous promoter lowered cytoplasmic Pi levels in the *mgtC* mutant, with no such effect observed on the vector control [10]. In another study investigating the target gene of virulence protein MgtC of *S. typhimurium*, MgtC targeted PhoR HK and activated PhoB-dependent gene transcription independent of cytoplasmic Pi levels through the determination of cytoplasmic Pi levels, without any difference between the *mgtC* mutant and wild type strains under low Mg^2+^ conditions [34]. In the aforementioned study of the activated PhoBR under low Mg^2+^ condition in *S. typhimurium*, the PhoU Arg184 residue interacted with PhoR HK domain [31]. The researchers further noticed a joint involvement of MgtC protein and PhoU Arg184 residue in activating the PhoBR system at low Mg^2+^ conditions [31], nevertheless, given a lack of experiments to characterize changes of cytoplasmic Pi levels, it was unclear whether cytoplasmic Pi was involved in the activation of PhoBR.

### Pi sensing

Pi is a constraint of the initial signal for gene transcription of Pho regulon. However, unlike other sensor kinases, PhoR cannot directly sense Pi owing to the absence of a periplasmic sensory domain [28]. The PhoU may assist PhoR to sense the Pi levels. In the study of *S. typhimurium*, the interaction of PhoU-PhoR PAS domains suppress the expression of Pho regulon genes in high-Pi environment [31]. It was previously thought that the PstSCAB-PhoU-PhoBR signaling pathway was activated by low extracytoplasmic Pi (Pi depletion in the medium) [2]. However, cytoplasmic Pi concentrations also had interference to the activity of PhoBR. In a recent study from *S. typhimurium*, the effect of cytoplasmic Pi on PhoBR activity was investigated by using alternative phosphorus sources and alternative phosphorus sources transporter independent of PhoB expression [9]. An artificially regulated *ugpBAECQ* operon (driven by an anhydrotetracycline-induced tetRA promoter) was constructed in their experiment, which decoupled Gly-3P metabolism from PhoB-independent transcription, where the *ugpBAECQ* locus encodes an ATP-dependent Gly-3P importer (UgpBAEC) and a cytoplasmic phosphodiesterase (UgpQ) capable of extracting Pi from Gly-3P. Results revealed that Gly-3P failed to metabolize in the absence of anhydrotetracycline, accompanied by deficient cytoplasmic Pi, and activated PhoBR, while in the presence of anhydrotetracycline, Gly-3P was metabolized and accumulated cytoplasmic Pi that inhibited PhoBR activity [9]. This evidence highlights that low cytoplasmic Pi condition can activate PhoBR in *S. typhimurium* in the context of unavailable exogenous phosphorus sources. Another study of *S. typhimurium* reported that PhoBR responded to low cytoplasmic Pi in low extracytoplasmic Mg^2+^ condition as mentioned above [10]. Besides, moderate levels of Pho could also activate PhoB by interacting with histidine kinase KinB, yet with relatively low activity of PhoR [35]. So far, there is insufficient data regarding the association of KinB with PhoB, and there is complex and sophisticated regulatory mechanism of Pi sensing, necessitating further elucidation and in-depth investigation.

## Bacterial virulence regulated by Pho regulon

Host-pathogen interactions can respond dynamically to diverse environmental conditions. A successful infection of the host by bacteria relies on the overcoming of various barriers and escape of recognition, requiring the participation of virulence factors in this process. Therefore, induction of virulence genes is pivotal in bacterial pathogenesis *in vivo*. The Pho regulon and bacterial virulence have been recognized to be closely connected. PhoB is implicated in the regulation of several other cellular processes and stress responses, including virulence trait expression, biofilm formation, secretion system, and quorum sensing.

### Escherichia coli

**Figure 2.**
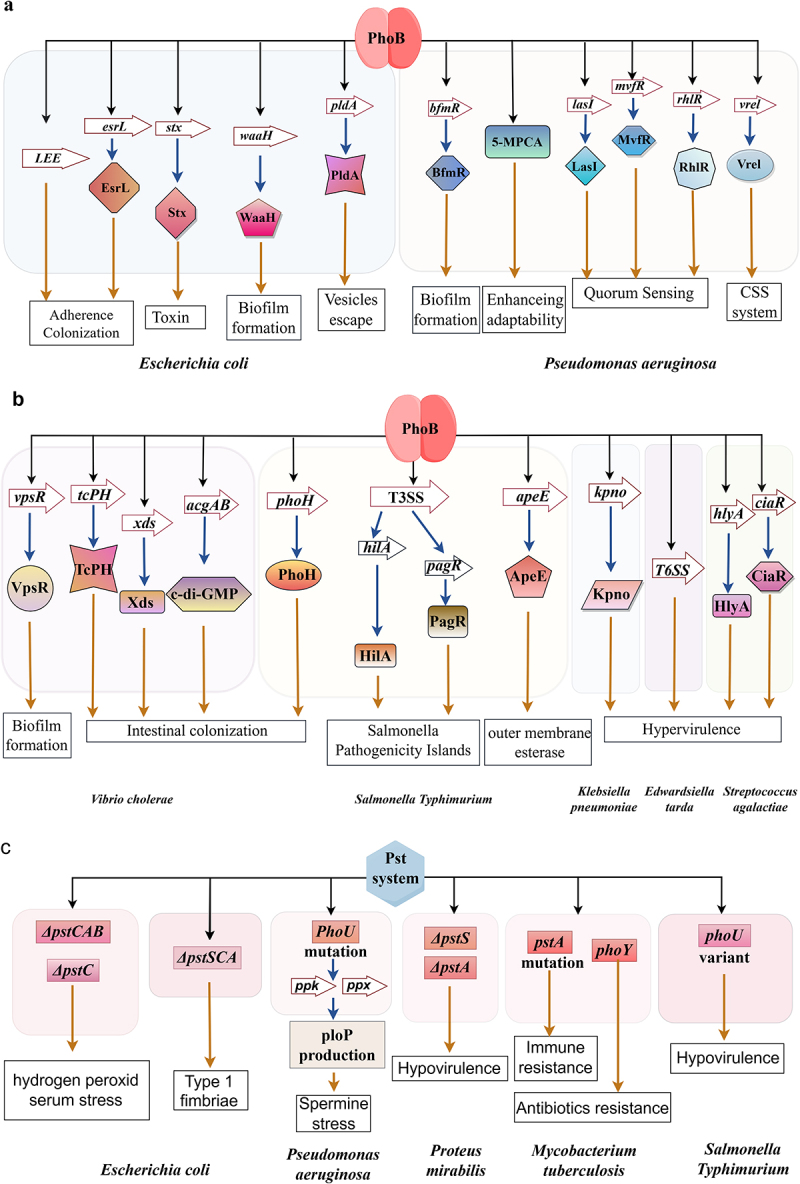
Pho regulon involvement in bacterial pathogenicity.

Enterohemorrhagic *E. coli* (EHEC) is a pathogenic Shiga toxin (Stx)-producing subgroup that can trigger diarrhea, hemorrhagic colitis, and hemolytic uremic syndrome. Stx, encoded by *stx*, is key for toxin production and EHEC diagnosis [36,37]. The LEE pathogenicity island mediates the attachment and effacement of lesions via bacterial adherence to enterocytes [38]. Both Stx and LEE are linked to the Pho regulon [39,40]. In EHEC O157:H7 EDL933, *LEE* expression and Stx toxin levels increased in the wild-type strain under Pi-limited environment, which, however, were downregulated after *phoB* deletion. PhoB was confirmed by electrophoretic mobility shift assay (EMSA) to bind *LEE* and *stx* promoter pho boxes (Figure 2a). Additionally, the small RNA EsrL, a regulator of the LEE pathogenicity island and *fimAICDFGH* operon (*fim*) in *E. coli*, was also related to the Pho regulon, and *esrL* expression would be directly repressed by PhoB under low-Pi condition (Figure 2a) [41].

(a) PhoB regulates the virulence of *E. coli*, *P. aeruginosa*. (b) PhoB regulates virulence of *S. typhimurium*, *V. cholerae, K. pneumoniae*, *E. tarda* and *S. agalactiae*. (c) Mutations in the PstSCAB system and auxiliary protein PhoU affect the virulence of *E. coli*, *S. typhimurium*, and *P. mirabilis*. The PstSCAB system is required for persistence and mediates resistance in *M. tuberculosis*.

Pho regulon has also been revealed to be associated with the formation of *E. coli* biofilms. The biofilms formed by bacteria can resist the attack of the host immune system and the penetration of antibiotics, leading to an estimated increase the resistance of bacteria to antibiotics, constituting a major cause of persistent clinical chronic infection. In the *E. coli* O157:H7 strain EDL933, the *ΔphoB* mutant formed significantly less biofilm under low-Pi condition [42], in contrast, the *ΔpstCAB* mutant exhibited a significant increase in biofilm formation compared to the wild-type strain, and the *ΔpstCABΔphoB* double mutant exhibited reduced biofilm formation in relative to the *ΔpstCAB* mutant [42], underscoring significantly increased biofilm formation owing to the complementation of the double mutant with *phoB*. Meanwhile, the expression of *waaH* was upregulated in the *ΔpstCAB* mutant strain, and based on transcriptomics data [42]. Moreover, PhoB could directly bind to the *waaH* promoter, as evidenced by data sourced from EMSA. Furthermore, with the construction of the *ΔwaaH* strain, the biofilm formed by the *ΔwaaH* mutant was significantly less than that of the wild-type strain. Simultaneously, the biofilm formed by the *ΔpstCABΔwaaH* strain was significantly less than that formed by the *ΔpstCAB* mutant [42]. Collectively, PhoB could be activated and participates in regulating *waaH*, highlighting the importance of PhoB in biofilm formation in the *ΔpstCAB* mutant. But in *E. coli K12 MC4100*, the activation of PhoB was mediated by acetyl Pho with high polyphosphate (polyP) levels in the stationary phase, negatively regulating biofilm formation [43]. Clearly, there is a contradiction in the regulation of biofilm formation by PhoB in *E. coli* O157:H7 strain EDL933 and *E. coli K12 MC4100*, which may be attributed to strain differences. In *E. coli* O157:H7 strain EDL933, the *ΔphoB* single mutant formed significantly less biofilm than the wild-type strain under low-Pi condition, while in *E. coli* K12 MC4100 strain, the deletion of *phoB* resulted in increased biofilm formation compared to the wild type in low-Pi condition.

In a study on urinary tract infection, UroPathogenic *E. coli* (UPEC) escaped fusiform vesicles in bladder cells related to PhoBR [44]. UPEC needed to escape fusiform vesicles featured by exocytosis in urinary tract infection. In their study, the outer membrane phospholipase PldA of UPEC could assist evasion through degrading vesicle membranes, and the Pho transporter PIT1 decreased Pi levels in the vesicles during infection of UPEC that activated PhoBR to upregulate PldA, facilitating membrane degradation and host evasion [44]. Current research on the role of the Pho regulon in bacterial virulence has primarily focused on the impact of Pho regulon mutations on virulence factor expression in vitro. However, few studies have investigated how the Pho regulon influences bacterial adaptation or host defense mechanisms under the low-Pi conditions that pathogens encounter during infection. If the studies similar to the PhoBR of UPEC assisted evasion from fusiform vesicles in urinary tract infections are designed, the understanding role of PhoBR in bacterial pathogenicity will be more comprehensive.

To sum up, as depicted in Figure 2a, PhoB may modulate toxin production, biofilm formation, and the adaptability of fusiform vesicles in bladder epithelial cells to affect the pathogenicity of *E. coli*.

### Pseudomonas aeruginosa

*P. aeruginosa* is a significant opportunistic pathogen capable of causing severe pneumonia [45], and its pathogenicity has been revealed to be closely associated with the type VI secretion system (T6SS) and quorum sensing (QS) system [46,47]. *P. aeruginosa* encodes three separate T6SSs (i.e. H1-, H2-, and H3-T6SS), which assist in pathogenesis by injecting effector proteins into the target cells [48–50]. QS is a communication mechanism that various virulence factors for pathogenic behaviors can be regulated through the activation of specific signals [51–54]. *P*. *aeruginosa* has four QS systems: *las*, *rhl*, *pqs*, and *iqs*, with the *las* system occupying the top hierarchical position to activate both *rhl* and *pqs* systems. Defects in low-affinity Pi transporter PitA would reduce intracellular Pi levels, thereby activating PhoB to upregulate H2-/H3-T6SS gene expression and activate the QS system in a PhoB-dependent manner, given a disappearance of the effect upon *phoB* deletion [55]. Meanwhile, *rhlR*, *lasI*, *pqsA*, and *mvfR* of QS system could be activated by PhoB under low-Pi condition, with the identification of PhoB-binding sites in the promoter regions of *rhlR*, *lasI*, *pqsA*, and *mvfR*, and PhoB could positively regulate *rhlR* expression, while this activation effect was abolished in *ΔphoB* mutant strains (Figure 2a) [56–58]. Besides, PhoB was identified to compete with LasR and RsaL to regulate *lasI* [58]. Critically, RsaL is a negative regulator of *lasI*, which can be activated by the *lasI* cognate regulator LasR [59]. The interaction of PhoB, MvfR-QS, and the pyoverdine iron acquisition system could activate a lethal phenotype of *P. aeruginosa* under Pi-depleted condition, and *P. aeruginosa* grown on low-Pi medium showed increased *C. elegans* mortality compared to high Pi medium [60].

The cell-surface signaling (CSS) is also an important regulatory system in *P. aeruginosa*, which can sense environmental signals and transmits them into the cytoplasm [61]. The PUMA3 CSS system, composed of a CSS-like receptor (VreA), anti-sigma factor (VreR), and sigma factor (σ^VreI^) [47,62], can exert potential regulatory effects on virulence factors in *P. aeruginosa*. PhoB can activate the expression of the *vreAIR* operon in a σ^VreI^-dependent manner under Pi-limited environment [63–65]. Noticeably, in both zebrafish embryos and a human alveolar basal epithelial cell infection model, the absence of *vreI* reduced Pi starvation-induced virulence [66].

The formation of biofilms of *P. aeruginosa* is associated with PhoB [67,68]. BfmR is a biofilm maturation regulator belonging to the BfmRS TCS, and PhoB was demonstrated to be required for BfmR to promote biofilm formation by *P. aeruginosa*, with significantly less biofilm formed in case of *ΔphoB* mutant (Figure 2a) [69]. The researchers identified through high-throughput sequencing and global transcriptome analyses that BfmR could bind to the promoters of multiple genes belonging to either the CzcR or PhoB regulon, or both.

In co-infection of *P. aeruginosa* and *Candida albicans* (*C. albicans*), *C. albicans* can produce ethanol to modulate the behaviors of *P. aeruginosa*, thus affecting the virulence of *P. aeruginosa* at sub-inhibitory concentrations [70,71]. For *P. aeruginosa*, ethanol has been found to stimulate the production and secretion of phenazine 5-methyl-phenazine-carboxylic acid (5-MPCA), which can enter *C. albicans* and react with basic amines to form a red pigment, thus triggering redox stress and death in *C. albicans* [72,73]. PhoB has been reported to be involved in the production of antifungal 5-MPCA by *P. aeruginosa* [74]. The production of ethanol and competition for Pho by *C. albicans* would further promote *P. aeruginosa* PhoB-dependent 5-MPCA production by *P. aeruginosa*, thus decreasing *C. albicans* fitness and increasing the competitive advantage of *P. aeruginosa* (Figure 2a).

In addition, PhoB can interact with TctD, an RR of the TctDE TCRS involved in sensing the availability of carbon sources, thus adapting to complex environments [57]. PhoB can also positively regulate the production of the pyocyanin toxin (a bioactive pigment that acts as a virulence factor and QS signaling molecule) of *P. aeruginosa* in Pi-limited environment [56]. During infection with *P. aeruginosa*, pyocyanin exposes host cells to oxidative stress, which may further cause damage to host tissues [75,76]. Furthermore, the *ΔphoB* mutant and double mutant *ΔpstSphoB* showed no bacterial swarming motility [77]. Swarming refers to a collective mode of motion in which bacteria migrate rapidly over surfaces, forming dynamic patterns of whirls and jets, leading to biofilm formation [78].

To conclude, PhoB can regulate the pathogenicity of *P. aeruginosa*, mainly involving in T6SS, QS system, and the formation of biofilms, as depicted in Figure 2a.

### Salmonella enterica serovar typhimurium

*S. typhimurium* is a major foodborne intracellular bacterial pathogen that contributes to the development of acute gastroenteritis in humans. In earlier research applying competitive infection assays with wild type strains, deletion of either *phoB* or *phoR* resulted in significant impairment of the bacterial replication within HeLa cells and RAW264.7 macrophages [79]. More recently, another study on *S. typhimurium* ST1120 showed substantially attenuated pathogenicity in the *ΔphoBR* mutant strain [80]. This was particularly evident in an animal infection model of BALB/c mice, where the mutant strain demonstrated a 1,000-fold higher LD50 (half-maximal lethal dose) compared to the wild-type strain in intraperitoneal challenge experiments, indicating severely compromised virulence [80]. Moreover, the *ΔphoBR* mutant offered protection against infection in the constructed BALB/c mouse model. Immunization with the *ΔphoBR* mutant further reduced the bacterial burden in mouse spleen and liver, increased the response of IgG and IgM antibodies, and enhanced the IgG2a/IgG1 ratio [80].

The pathogenicity of *S. typhimurium* depends largely on two type 3 Secretion Systems (i.e. T3SS1 and T3SS2), which are encoded by two distinct genetic loci, Salmonella Pathogenicity Islands 1 and 2 (SPI1 and SPI2). Regulation of SPI1 involves HilA, HilC, HilD, HilE, and RtsA transcriptional master regulators. A prior study showed that *hilA* genes were repressed by the PhoBR (Figure 2b), and it was speculated that gene products in the Pho regulon or PhoB would be responsible for activating the expression or activity of a repressor of *hilA* expression [81]. In another study, HilC and HilD were discovered to promote the regulation of *hilA* expression in *S. typhimurium*, and that study proposed a hypothesis that PhoB might modulate the expression or activity of *hilC* or *hilD* to regulate *hilA* expression [82]. Interestingly, *phoH* of the Pho regulon is recently found to be regulated by the HilD sequence in *S. typhimurium*, independent of PhoBR. *PhoH* is induced by PhoBR TCRS in *E. coli*, which, however, is poorly understood regarding its function, although it shares similarity to a family of helicases in aspects of ATPase activity and sequence. In the same study, the *ΔphoH* mutant demonstrated a slight decrease in intestinal colonization of *S. typhimurium* compared to the wild-type [83]. Beyond the above, PhoB also induced PagR, a novel regulator, in low-Pi condition at the stage of post-infection of macrophages, which responded to the low Mg^2+^ and low Pi signals, regulating *SPI-2* expression in *S. typhimurium* during the entire period of intramacrophage growth (Figure 2b) [84]. The mutual regulation between the PhoBR and T3SS may be a key player in the infection of *S. typhimurium*, pending further study to elucidate the specific molecular mechanism. Additionally, the expression of *apeE*, a gene encoding an outer membrane esterase, in *S. typhimurium* was induced in Pi-limited environment and regulated by PhoBR [85].

Besides, the Pho regulon of *S. typhimurium* also exhibits a relationship with metabolic processes [18]. According to an investigation using proteomic profiling in Pi starvation, a total of 389 bacterial proteins were differentially regulated in *S. typhimurium* upon the shift from Pi-rich to Pi-low conditions, with upregulation found for glycolysis, pentose Pho pathway, pyrimidine degradation, glycogen, and trehalose metabolism of metabolic processes [18]. With further construction of the deletion strains of *phoB*, the same research group continued to analyze the proteomic changes of the wild type strains in low-Pi condition, with the identification of two key enzymes for bacterial N-acetylglucosamine catabolism NagA and NagB, depending on PhoB regulation under Pi-limited condition [18]. Moreover, PhoB was required for the full activation of NagB in response to low Pi, as revealed by immunoblotting and β-galactosidase assays (Figure 2b) [18].

PhoB is associated with the regulation of Salmonella pathogenicity islands genes and the enzymes related to the metabolic of N-acetylglucosamine. The absence of PhoB can reduce pathogenicity in *S. typhimurium*, as shown in Figure 2b.

### Vibrio cholerae

Cholera is a *V. cholerae*-induced potent intestinal infectious disease, accompanied by severe watery diarrhea. In *V. cholerae*, compared to the wild type, the *phoB* mutant of *V. cholerae* O1 might impair the ability to colonize in adult rabbit ligated ileal loop assays [86,87]. The deletion of *phoB*, might affect the expression of *vpsR*, a positive regulator of biofilm formation, resulting in significantly enhanced biofilm formation under Pi-limited condition [88]. PhoB might also regulate the expression of *acgAB*, a gene encoding the second messenger cyclic di-GMP (c-di-GMP) metabolic enzymes, thereby influencing infant mouse small intestine infection at late stages [89]. PhoB could negatively regulate the expression of *tcpPH*, which is essential for the colonization of the small intestine [90]. Furthermore, PhoB was identified as the dominant activator of *xd*s expression by Tn-seq in *V. cholerae*, and the deletion of *phoB* would reduce the expression of *xds* [91]. The *xds* gene encodes the nuclease Xds, which can mediate the escape from neutrophil extracellular traps during intestinal infection, thus assisting in evading the innate immune response and promoting survival. Moreover, Xds might contribute to the persistence and infection of *V. cholerae*, as it can control the three-dimensional biofilm formation and bacterial detachment from biofilms via degradation of extracellular DNA [92].

In addition, in *V. cholerae* N16961 cells, an El Tor biotype, the *ΔphoB* mutant was less resistant to H_2_O_2_ challenge than wild-type cells in Pi-limited medium; and it could accumulate more intracellular reactive oxygen species (ROS) in Pi-limited medium than in Pi-sufficient medium [93]. Recognizing as a pivotal strategy in host-mediated pathogen clearance [94], ROS, comprising superoxide (O_2_•-), hydrogen peroxide (H_2_O_2)_, hydroxyl radical (•OH), and singlet oxygen ((1)O_2_) [94,95], may induce oxidative stress when accumulated, causing damage to nucleic acids, proteins, lipids, etc [96]. It has been documented that catalases KatG and KatB can respond to oxidative stress in *V. cholerae* [95], but PhoB is not involved in regulating *katB* and *katG* gene expression in Pi-limited environment [93]. Therefore, the PhoB may be a critical protective factor against oxidative stress through other mechanisms than catalase regulation in *V. cholerae* N1696.

With respect to the above, the PhoB can affect the pathogenicity of *V. cholerae* by interfering biofilm formation, the ability of colonization and oxidative stress resistance (Figure 2b).

### Klebsiella pneumoniae

*K. pneumoniae* is a notorious nosocomial pathogen that has been confirmed to be the chief culprit of various infections such as septicemia, pneumonia, urinary tract infections, and trauma-related infections. KpnO is one of its outer membrane proteins (OMPs), accepting as a major virulence factor to regulate the efficacy of antimicrobial treatment. KpnO may participate in influencing the production of capsular polysaccharides, a crucial virulence factor for host interactions [97]. PhoBR has been found to be a potential regulator of kpnO, with a PhoB-binding site detected upstream of kpnO [97]. Loss of PhoBR-regulated kpnO may result in increased antimicrobial resistance, heightened susceptibility to gastrointestinal stress, and reduced virulence of *K. pneumoniae* NTUH-K2044 (Figure 2b) [97].

### Edwardsiella tarda

*E. tarda* from the Enterobacteriaceae family may occasionally lead to gastroenteritis and bacteremia in humans, in addition to causing infections in fish typically [98,99]. In *E. tarda* PPD130/91, PhoBR and iron-sensing coding proteins could regulate virulence genes associated with T6SS (Figure 2b) [19], a crucial virulence factor in *E. tarda* [100,101]. Deletions of T6SS *evpB* and *evpC* were reported to decrease the virulence in blue gourami hosts, and mutations in *evpABC*, *evpEFGHI*, *evpKLMNO*, and *evpP* might also cause attenuated virulence [101,102]. In *E. tarda* PPD130/91, PhoB could regulate the transcription of T6SS genes by binding to the secretion regulator EsrC on the promoter of *evpA* [19].

### Streptococcus agalactiae

*Streptococcus agalactiae* (*S. agalactiae*) has been discovered to be a major inducer of puerperal sepsis and neonatal meningitis in pregnant women. It is parasitic in the maternal genital tract and may cause infection in the fetus, leading to potential postpartum infection and bacteremia. Recently, the potential role of PhoBR TCRS has been confirmed in *S. agalactiae*. The deletion of *phoB* would increase biofilm thickness, and PhoB could directly bind to the promoter regions of *hemolysin A* and *ciaR* through lacZ reporter and bacterial one-hybridization [103]. In addition, in the *S. agalactiae* TOS01 strain, the *phoB* mutant showed a lower adherence and invasion rate, which was less virulent than the wild-type strain. Besides, *ΔphoB* strain through intraperitoneal injection exhibited a 93.1% survival rate after challenge with TOS01 in golden pompano (Figure 2b) [104].

## Pst system and bacterial virulence

Type 1 fimbriae are a key virulence factor required to establish infection in *E. coli* [105–108]. In the UPEC strain CFT073, the Pst system was found to induce the formation of type 1 fimbriae [109]. The *ΔpstSCA* mutant was deficient in the production of type 1 fimbriae when compared to the wild-type strain and *ΔpstSCA* complemented strains (Figure 2c). Type 1 fimbriae are encoded by the *fim* operon that is controlled by a phase-variable promoter (*fimS*). Furthermore, the *ΔpstSCA* mutant had significantly lower activation state of the *fim* promoter than that of the wild type and the *ΔpstSCA* complemented strains in LB broth, coupled with reduced expression of the recombinases *FimB*, *IpuA*, and *IpuB* that promote the inversion of *fimS*. In the murine UTI model, the transcription of *fimS* was downregulated in the *ΔpstSCA* mutant at 24 h and 48 h, while less colonization in the mouse urinary tract was observed in the *ΔpstSCA* mutant compared to the wild-type strain. In another study, the diguanylate cyclase encoded by *yaiC* was recognized to connect the Pst system and type 1 fimbriae formation in the UPEC strain CFT073 [110]. Deletion of *yaiC* in the *ΔpstSCA* mutant restored type 1 fimbriae production by increasing the expression of *fim* structural genes. In avian pathogenic *E. coli* (APEC), *ΔpstCAB*, *ΔpstC*, and *ΔphoR* damaged to type 1 fimbriae and affected other virulence attributes, including reduced resistance to the bactericidal effects of rabbit serum and more resistant to H2O2 in LB agar plates [111]. In addition, the *ΔphoB* and *ΔpstCΔphoB* mutants exhibited an increased sensitivity to H2O2 in low-Pi agar plate compared to wild-type strains [111]. APEC is an extra-intestinal pathogenic *E. coli* (ExPEC), which has been recognized to be one of the leading causes of mortality and morbidity in poultry, causing diverse local and systemic infections in avian species [107,112]. In addition, insertional mutations in *pstC* and *pstA* led to the loss of biofilm formation by *Pseudomonas aureofaciens* PA147–2, and the *ΔpstS* mutant was defective in biofilm formation in *Proteus mirabilis* (*P. mirabilis*) [113]. In *P. mirabilis*, *pstS* or *pstA* mutation would significantly reduce the infectivity in the urine, bladder, and kidneys of mice. Furthermore, the general growth defect was responsible for the attenuation of PstSCAB [114]. In *Mycobacterium tuberculosis* (*M. tuberculosis*), PstA was confirmed to be required to resist IFN-γ-dependent immunity and to promote the persistence of *M. tuberculosis* in the host [115]. The absence or mutation of the Pst system would mimic a low Pho state, where PhoB remained continuously activated even in the presence of sufficient environmental Pho, leading to a constitutive expression of the Pho regulon [42]. Given a pleiotropic effect, the over-activation of PhoB may be involved in the regulation of virulence genes, either directly or indirectly. However, there was a lack of constructing *phoB* deletion mutants to compare their pathogenicity with *pst* mutants in the existing studies, associated with poor understanding of the pathogenicity of complementation strains. So, there is still no further identification on target genes regulated by PhoB in the pathogen, whether they include virulence-related genes. Anyway, it is a great challenge to determine the role of PhoB in the pathogenicity of these *pst* mutants, necessitating further research for clarification.

## PhoU and bacterial virulence

In *S. typhimurium*, the *ΔphoU* variant strain showed decreased replication within macrophages, and *ΔphoU* strains were completely defective in mice compared to the wild-type strain (Figure 2c) [31].

Putrescine, spermidine, and spermine are the three most common polyamines. Specifically, excess spermine may be harmful for cell growth. *P. aeruginosa* possesses six γ-glutamylpolyamine synthetases (GPSs) encoded by the *pauA1-pauA7* to initiate polyamine catabolism [116]. The Pho regulon and polyP synthesis might be triggered by the harsh effects of spermine, and in the absence of *pauA2*, a spermine modification enzyme gene, spermine-resistant strains exhibited the activation of the Pho regulon [117]. The *phoU* mutation was found to differentially impact the expression of polyP kinase *ppk gene* and exopolyphosphatase *ppx* gene contributing to the accumulation of polyp granules [118]. Accumulation of polyp granules can resist the toxicity of spermine [117]. The *phoU* mutation could also affect the growth, mobility (swimming, swarming, and twitching), and rhamnolipid synthesis [117].

In *Staphylococcus aureus* (*S. aureus*), PhoU2, the PhoU homologs, also has established association with virulence. α-hemolysin is an exotoxin and one of main virulence factors in *S. aureus*. The *ΔphoU2* variant was detected, via western blot, with enhanced α-hemolysin activity and higher α-hemolysin levels; besides, intracellular survival assay revealed that it could decrease the replication in the human lung epithelial cell line A549. In addition, in another assay assessing the tolerance of *ΔphoU2* to stresses [sodium dodecyl sulfate (SDS) and H_2_O_2_], the *ΔphoU2* variant resulted in elevated sensitivity to H_2_O_2_ and SDS of *S. aureus* [119].

## Pho regulon response to stress

Pho starvation can activate the expression of the Pho regulon genes and trigger a stress response. RpoS is a sigma factor in the stable period that can regulate stress response and the growth of bacteria against environmental stress [120]. Genetic evidence has indicated the existence of complementarity in the intergenic region between *pstA* and *pstB* to the untranslated leader region of *rpoS*. The small intergenic region of the 3’ end of the *pstA* transcript could interact with the untranslated leader region of *rpoS* mRNA, further accelerating the accumulation of RpoS under Pi-limited condition facilitated by Hfq [121]. In *V. cholerae* O1, PhoBR was required for *rpoS* expression of the stationary-phase cells [122]. But the study showed that the *phoBR* mutants exhibit higher tolerance than wild-type strains under thermal and hyperosmolar pressure, which suggested that *V. cholerae* cells can use different strategies to effectively respond to specific stress stimuli in the absence of *phoBR* and RpoS-regulated gene products condition [122].

## Pho regulon and modification of cell surface components

In general, the Pho regulon consists of three members of PhoBR, PstSCAB, and PhoU. Lipopolysaccharide (LPS) is a unique component of the cell wall of gram-negative bacteria, functioning significantly in maintaining the integrity of the outer membrane and bacterial viability, providing a permeability barrier function. They are composed of three regions (i.e. lipid A, oligosaccharide core, and O-specific polysaccharide), and lipid A is the bioactive center and major toxic component of LPS [123]. The hexa-acylated 1-pyrophosphate lipid A was observed to be reduced in *ΔpstCAB* mutant of ExPEC strains compared to wild-type [124]. Through microarray analysis, LPS-related genes were found to be downregulated in the *ΔpstCAB* mutant compared to wild-type, with a pho box identified upstream of LPS biosynthesis-related genes. For example, *rfaJ*, which encodes an LPS 1,2-glucosyltransferase, was discovered to be involved in LPS core biosynthesis. The *rfaP* and *rfaY* genes were both involved in LPS inner-core phosphorylation, and the *eptA* gene participated in the pEtN covalent modification of lipid A [124]. The same study also reported that compared to wild-type strain, the *ΔpstCAB* mutant exhibited higher susceptibility to polymyxin B, cecropin P1 and vancomycin, while these phenotypes would be restored through the complementation of *ΔpstCAB* mutation [124]. However, further researches are needed to confirm whether these phenotypic changes are caused by the reduction of hexaylated 1-pyrophosphate lipid A in the *ΔpstCAB* mutant, or the activation of PhoB in the *ΔpstCAB* mutant regulates LPS biosynthesis genes. As discussed in the pst system and virulence, mutations in this system often affect bacterial virulence among different strains. Given that further investigation on bacterial surface modifications in *pst* system mutant was absent in this study, it remains unclear and necessitates further studies to clarify whether the observed virulence changes caused by *pst* system mutations are associated with the alterations of bacterial surface components.

## Pho regulon mutations and bacterial antibiotic resistance

In *K. pneumoniae* NTUH-K2044, compared to the wild type strain, the *ΔphoB* mutant showed decreased minimal inhibit concentration (MIC) for amikacin, cefepime, ceftazidime, chloramphenicol, colistin, erythromycin, streptomycin, and trimethoprim [97]. In *M. tuberculosis*, detection after treatment with CIP-EMB and RIF-EMB (ciprofloxacin [CIP] and ethambutol [EMB], rifampin [RIF], and EMB) revealed that lower survival rate of the *ΔphoY1ΔphoY2* (the *phoU* orthologs of *E. coli*) than the wild type [125]. In the MIC assay, the RIF MIC_90_ of *ΔphoY1ΔphoY2* mutant was lower than that of the wild type; moreover, RIF treatment improved the clearance of *ΔphoY1ΔphoY2* mutant than wild-type in mouse infection model [125], suggesting potential link between the Pho regulon and bacterial antibiotic resistance. However, so far, it is still unclear whether the observed resistance changes are directly mediated by the Pho regulon or arise indirectly from secondary effects, which require further studies given the pleiotropic effect of mutations in these genes.

## Conclusion and perspectives

**Figure 3.**
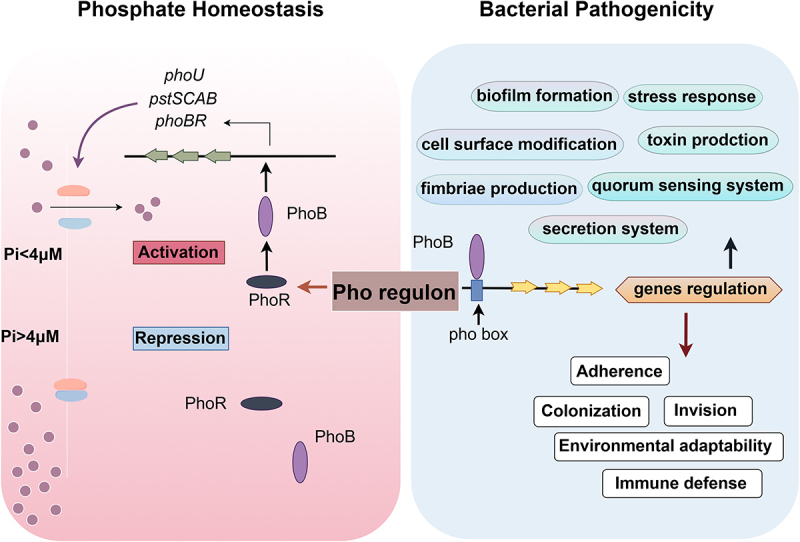
Pho regulon connects Pho homeostasis with bacterial virulence.

Environmental adaptation and virulence regulation are essential for bacterial infections. Both can determine the viability, transmission efficiency, and pathogenicity of bacteria in the host. The PhoBR TCRS of the Pho regulon is an important signal transduction pathway in bacteria that integrates environmental signals, regulates gene expression, and alters bacterial physiological behavior. It is a global regulatory network that connects Pho homeostasis and bacterial pathogenicity (Figure 3). PhoB serves as a central transcription factor that controls various virulence genes by binding to the pho box located upstream, influencing bacterial pathogenicity, and promoting infection. Mutations in the PstSCAB system also affect virulence; however, this attenuation can be attributed to the constitutive activation of PhoB. The formation of biofilms and secretion systems is widely influenced by different bacteria. They are essential for pathogenic bacterial processes.

However, it seems that there are some ambiguous areas in the regulation of virulence by the interaction of PhoB with the pho box in virulence genes. Previous studies have focused on changes in virulence deformation of knockout strains *in vitro*. However, there is limited research on the differential responses of bacterial subpopulations with PhoBR in various microenvironments of the host of low Pho and whether it plays a role in host microenvironment adaptation or defense responses. Future studies may utilize genetically encoded fluorescent sensors (like FRET probes) to monitor the dynamics of PhoB activity in real time, combined with microfluidic simulations of host environments (such as fluctuations of Pho in the intestine and within macrophages), along with single-cell transcriptomics/proteomics to uncover the heterogeneity regulated by PhoBR, and spatial transcriptomics to analyze the spatial correlation of bacterial PhoBR activity and interactions with host cells in infected tissues. Additionally, organoid chip infection models can simulate the Pho microenvironment of human tissues (such as lungs and intestines) to investigate the role of PhoBR in real infection scenarios. Dual RNA-seq can be used to synchronize the analysis of the activation of bacterial PhoBR during infection and its correlation with host immune responses. Elucidating the environmental adaptation and subsequent virulence regulation of the Pho regulon in host cells may facilitate the development of new antimicrobial strategies.

## Data Availability

Data sharing is not applicable to this review article as no new data were created or analysed in this study.
